# Effect of carbonated water on cerebral blood flow in the frontal region: a study using near-infrared spectroscopy

**DOI:** 10.3389/fnbeh.2024.1409123

**Published:** 2024-12-10

**Authors:** Wataru Kosugi, Brian Sumali, Nozomu Hamada, Yasue Mitsukura

**Affiliations:** ^1^School of Integrated Design Engineering, Keio University, Yokohama, Japan; ^2^Faculty of Science and Technology, Keio University, Yokohama, Japan

**Keywords:** cerebral blood flow, carbonated water, frontal region, near-infrared spectroscopy, oxyhemoglobin

## Abstract

**Introduction:**

Carbonated water (CarbW) affects the swallowing function associated with the action of the brainstem. In addition, CarbW ingestion promotes mean blood flow in the middle cerebral artery, which is associated with blood flow to the frontal and temporal lobes. In this milieu, studies regarding the effect of drinking CarbW on brain activity are of significance. In the present study, we compared the changes in cerebral blood flow in the frontal region before and after the ingestion of CarbW or uncarbonated water (SW).

**Methods:**

Near-infrared spectroscopy was used to continuously measure the cerebral blood flow at 22 channels in the frontal region of 13 healthy young adults for 10 min before and after the ingestion of CarbW or SW. We statistically compared the changes in oxyhemoglobin concentration before and after the ingestion of CarbW or SW.

**Results:**

Compared with that before CarbW ingestion, the oxyhemoglobin concentration in the left frontal region increased after CarbW ingestion. In particular, a significant increase (*p* < 0.05) was observed in the ch21 region. On the contrary, no marked increase or decrease in cerebral blood flow was observed after SW ingestion compared with that before ingestion.

**Discussion:**

The activated part of the frontal region (ch21) corresponds to the vicinity of the orbitofrontal cortex, which is reportedly activated by rewarding stimuli. In addition, as the orbitofrontal cortex is located at the terminal end of the reward pathway of the mesocortical system, CarbW ingestion might have acted on the dopaminergic reward pathway of the mesocortical system.

## 1 Introduction

Ingestion of carbonated water (CarbW) reportedly affects the swallowing function (Barry and Regan, 2021; Larsson et al., 2017; Michou et al., 2012; Min et al., 2022; Miura et al., 2009; Morishita et al., 2014; Sdravou et al., 2012). In addition, CarbW induces the swallowing reflex by acting on the capsaicin-sensitive receptor (TRPV1) in the oral cavity and pharynx (Roper, 2014; Simons et al., 2019; Tsuji et al., 2020). CarbW stimulates the swallowing center of the medulla oblongata, located in the brainstem. Furthermore, ingestion of CarbW promotes the mean blood flow in the middle cerebral artery, which is associated with the blood flow in the frontal and temporal lobes (Fujii et al., 2022). Hence, it is important to investigate the effect of ingesting CarbW on brain activity. Therefore, in the present study, we compared the changes in cerebral blood flow in the frontal region before and after the ingestion of CarbW or uncarbonated water (SW). This study provides novel insights into the effect of CarbW ingestion on brain activity in the frontal region.

## 2 Materials and methods

### 2.1 Participant demographics

This study was approved by the Keio University Bioethics Committee (approval no: 2021–20). The recruitment period was from 1 September 2021 to 31 January 2022. Originally, 16 (12 male and 4 female) healthy university students voluntarily participated in this study. Written informed consent, including consent to participate and publish the findings, was obtained from all study participants. All participants were of Asian descent (100%), with 23.1% being female. The average age of participants was 22.1 ± 1.0 years.

### 2.2 Experimental procedure

**Figure 1:**
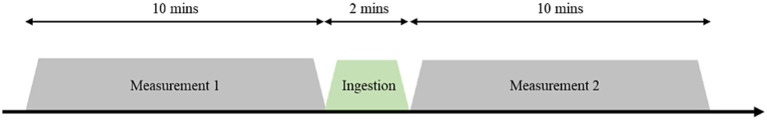
Measurements for the experiment. The participants were monitored for 10 min before and 10 min after drinking each sample (excluding the drinking time of 2 min).

In this study, the cerebral blood flow dynamics of the participants were recorded before and after they drank 200 mL of CarbW or SW. The experiments were started at 10:00 a.m. and conducted indoors in a room maintained at 20°C. The participants were monitored for 10 min before and 10 min after drinking each sample (excluding the drinking time of 2 min) as they rested with their eyes closed (Figure 1). Thirteen (10 male and 3 female) healthy young adults in their 20s completed the experimental procedures.

This study used a randomized crossover design: The order in which each participant completed their two trials (i.e., CarbW vs. SW) was randomly assigned. To ensure experimental accuracy, only one person underwent testing per day.

**Figure 2:**
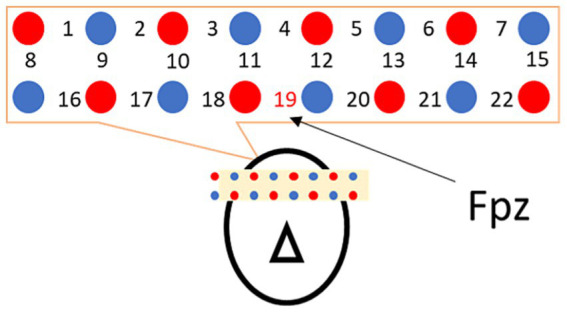
Probe arrangement. Light emitters and photodetectors are marked with red and blue circles, and the measurement points are indicated with numbers. For the frontal region, ch19 was set to Fpz, according to the International 10–20 system for assuring minimum between-subject position variability.

Cerebral hemodynamics were recorded using LIGHTNIRS from SHIMADZU Co. for near-infrared spectroscopy to identify regions innervated by cranial nerves. Probes (optodes) were arranged with respect to landmark *Fpz* in the International 10–20 system and placed at 22 sites above the frontal brain at a sampling frequency of 7.5 Hz (ch1–22; Figure 2).

The LIGHTNIRS system by Shimadzu is a portable functional near-infrared spectroscopy (fNIRS) device designed for research applications, such as monitoring brain activity by measuring variations in oxygenated (Oxy-Hb), deoxygenated (Deoxy-Hb), and total hemoglobin (Total-Hb). The device uses a 3-wavelength near-infrared semiconductor laser and an avalanche photodiode detector. Its compact dimensions are W253 × D222 × H68 mm, with a weight of approximately 1,600 g. It can handle up to 22 measurement channels and operates between 15 and 30°C in humidity levels of 45–85% (Light source: 3-wavelength near-infrared semiconductor laser, Class 1 [IEC-60825-1 (2007)]).

Before the experiment, we practiced attaching the probes to the designated positions 10 times to ensure consistency. During the experiment, measurements were taken three times with the same participants, and the average data were used.

Regarding cerebral blood flow measurement, three types of density data can be acquired: oxyhemoglobin, deoxyhemoglobin, and total hemoglobin concentrations. Total hemoglobin concentration is the sum of oxyhemoglobin and deoxyhemoglobin concentrations. For our study, oxyhemoglobin data were selected for analysis because changes in the oxyhemoglobin concentration are the most prominent. In each trial, changes in the oxyhemoglobin concentration were measured for 10 min before the participants started drinking the water and again for 10 min after they finished drinking (i.e., 2 min later).

### 2.3 Analysis of cerebral blood flow dynamics

Cerebral hemodynamics were analyzed according to the following procedure. Signal data of the change in cerebral blood flow are indirectly estimated by measuring the change in absorption due to changing oxyhemoglobin and deoxyhemoglobin concentrations using the fNIRS. The obtained signal data were first preprocessed to remove noise artifacts owing to the power supply by applying a low-pass filter with a cutoff frequency of 1 Hz. Next, hemoglobin concentration data were decomposed into systemic and functional (brain) components using a method for separating hemodynamic signals. This method can be represented by the following equation (Yamada et al., 2012):


$$\left(\begin{array}{c} \Delta HbO_{F}\\ \Delta HbR_{F} \end{array}\right)=\frac{1}{k_{F}-k_{S}}\left(\begin{array}{cc} -k_{S} & 1\\ -k_{F}.k_{S} & k_{F} \end{array}\right)\left(\begin{array}{c} \mathrm{\Delta HbO}\\ \mathrm{\Delta HbR} \end{array}\right)\text{;}$$

where *O* is the observed concentration of oxyhemoglobin, *R* is the observed concentration of deoxyhemoglobin, and $k_{F}$ and $k_{S}$ are proportionality constants.

$k_{S}$ describes a brain property called neurovascular coupling, where oxyhemoglobin increases and deoxyhemoglobin decreases as regional cerebral blood flow adjusts during neural activation. As such, $k_{F}$ (−1 < $k_{F}$ < 0) is defined as a proportional constant, establishing a proportional relationship between functional oxyhemoglobin and deoxyhemoglobin.

$k_{F}$ represents changes in the systemic component that lead to the dilation of blood vessels. In this process, both oxyhemoglobin and deoxyhemoglobin levels increase. Therefore, $k_{S}$ (0 < $k_{S}$) is defined as a proportional constant, establishing a proportional relationship between systemic oxyhemoglobin and deoxyhemoglobin.

Next, as the acquired data represented the degree of change with respect to the baseline, signals were baseline-corrected by setting the oxyhemoglobin concentration to 0 at the starting point of each recording. Fluctuations in the oxyhemoglobin concentration were signal-averaged separately at each of the 22 recording sites (ch1–22). Finally, this quantity was compared with that before ingestion at each channel to identify the brain regions that were functionally activated by the stimulus.

### 2.4 Statistical analysis

Data were analyzed using MATLAB R2021a software (The MathWorks, Inc.). Distributions of all variables were inspected using histograms, q–q plots, and the Shapiro–Wilk tests before conducting statistical analyses. As non-parametric data, the Wilcoxon signed-rank test was used for statistical comparisons. The significance level was set at a *p*-value of <0.05, and the false discovery rate (FDR) was used to control for multiple comparisons.

## 3 Results

**Figure 3:**
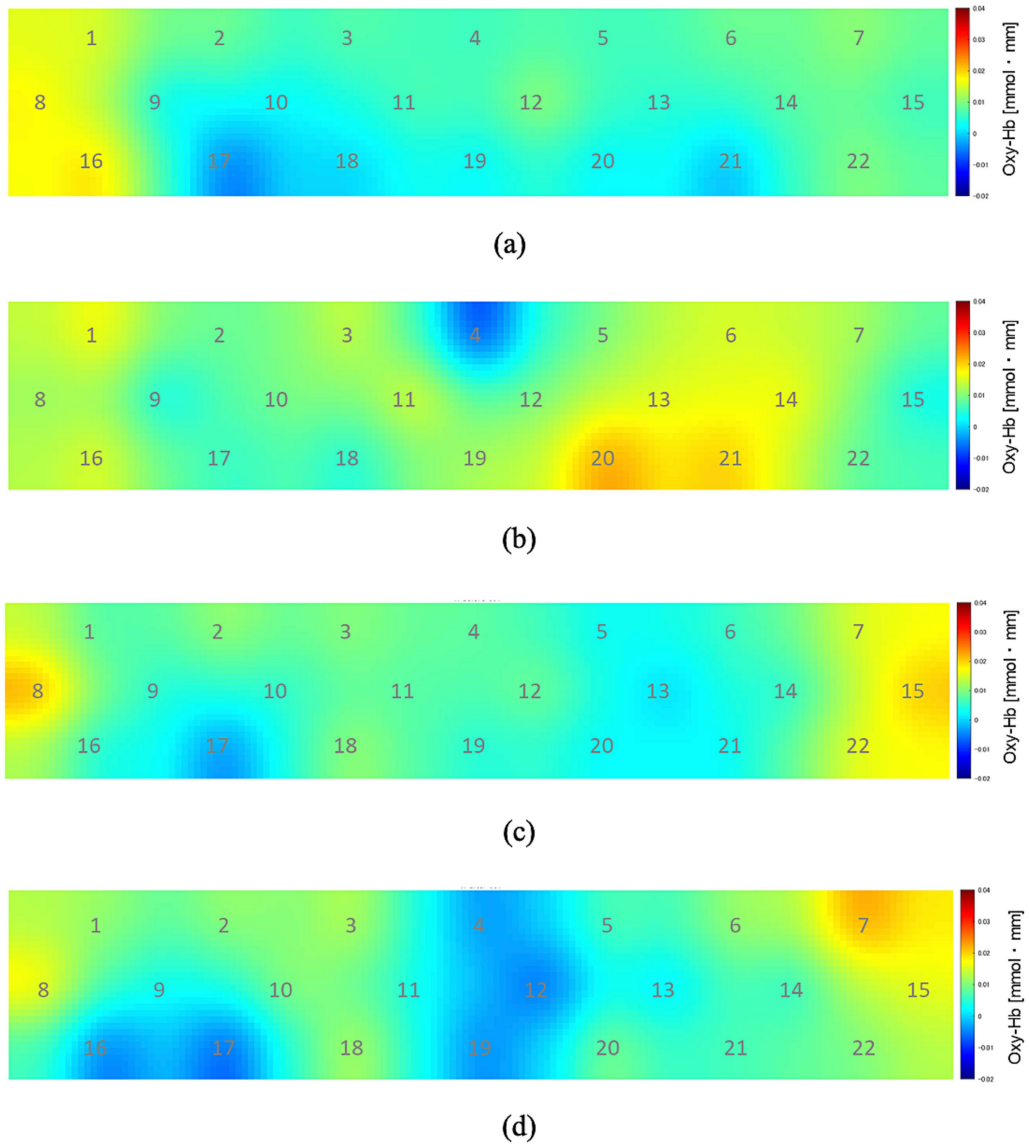
Oxyhemoglobin concentration at each recording channel (ch1–22). **(A)** During the 10 min before the participants drank carbonated water. **(B)** During the 10 min after the participants drank carbonated water. **(C)** During the 10 min before the participants drank uncarbonated water. **(D)** During the 10 min after the participants drank uncarbonated water.

**Figure 4:**
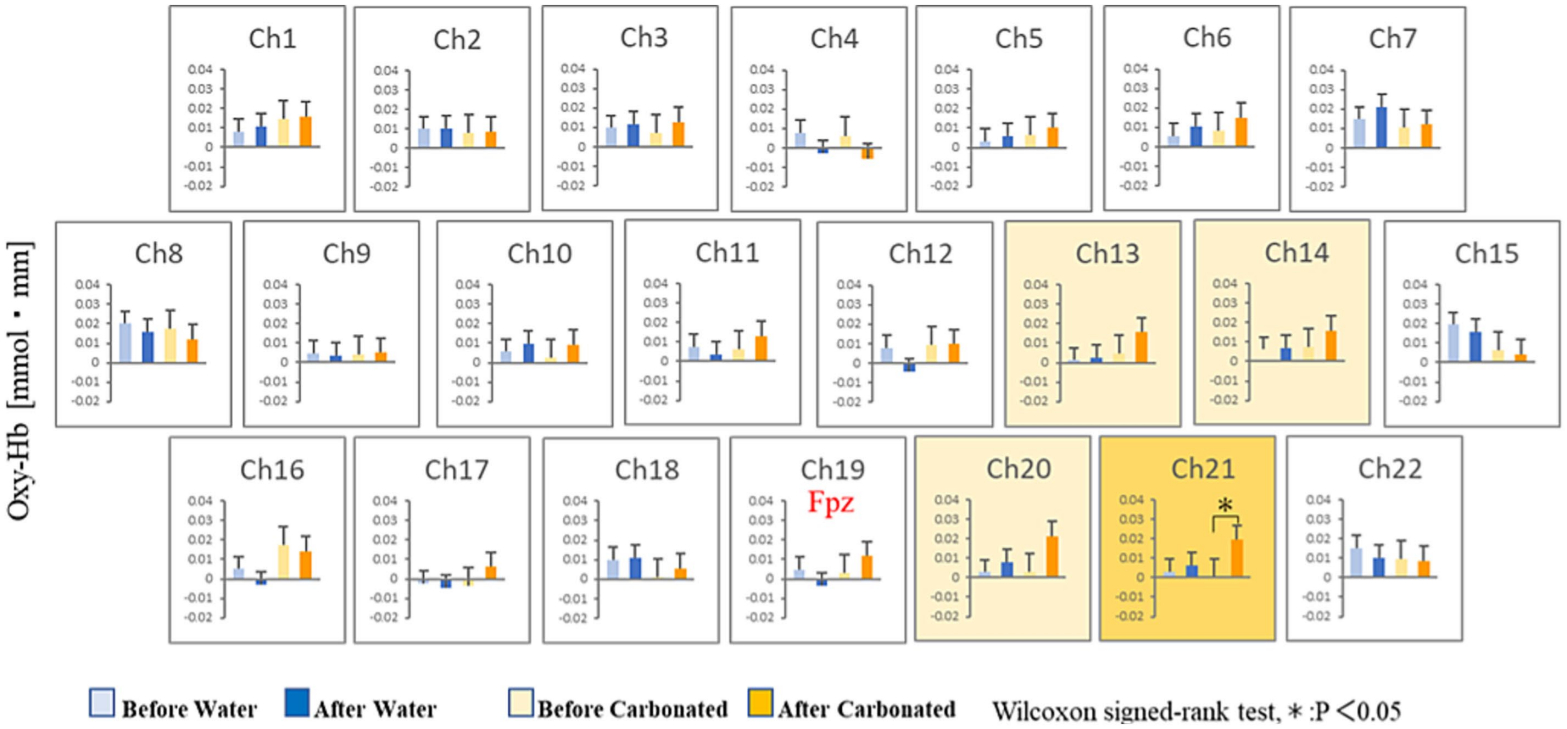
Statistical comparisons of oxyhemoglobin concentrations at each channel (ch1–22). The oxyhemoglobin concentrations before and after ingestion are compared.

Figure 3 shows the changes in oxyhemoglobin concentration observed at each recording channel (ch1–22) during the 10 min before and 10 min after the participants drank CarbW and SW. A comparison of the oxyhemoglobin concentration at each channel (ch1–22) before and after ingestion is presented in Figure 4.

Compared with that before ingestion, the mean oxyhemoglobin concentration during the 10 min after the ingestion of CarbW increased at the measurement regions of ch13, ch14, ch20, and ch21. In addition, a significant increase in oxyhemoglobin concentration was observed at the measurement region of ch21 after the ingestion of CarbW compared with that before ingestion. On the contrary, no significant difference in oxyhemoglobin concentration was observed before and after the ingestion of SW at any measurement region.

## 4 Discussion

The present study showed that, upon ingestion of equal amounts of CarbW and SW, marked changes in cerebral hemodynamics in the frontal region were observed after the ingestion of CarbW. Previous studies have reported that peripheral stimulation by CarbW induces swallowing movement, which suggests that CarbW acts on the region of the brainstem that controls the swallowing center. The hemodynamic changes caused by CarbW ingestion in this study suggest the effect of CarbW on the frontal region, which has a higher brain activity level than the brainstem. Furthermore, CarbW ingestion promotes mean blood flow in the middle cerebral artery. As the middle cerebral artery is also involved in the blood flow to the frontal region, CarbW ingestion may affect blood flow to the frontal region, which might have been the reason for the hemodynamic changes in the frontal region upon CarbW ingestion.

On the other hand, previous studies using human fMRI have shown a positive correlation between the value of reward and the fMRI signal of the orbitofrontal cortex (Breiter et al., 2001; O'Doherty et al., 2002; Daw et al., 2006; Kim et al., 2006; Tom et al., 2007; Plassmann et al., 2008; Kahnt et al., 2010). The activated part of the frontal region (ch21) corresponds to the vicinity of the orbitofrontal cortex, which is reportedly activated by rewarding stimuli (Walter et al., 2005). In addition, as the orbitofrontal cortex is located at the terminal end of the reward pathway of the mesocortical system, CarbW ingestion might have acted on the dopaminergic reward pathway of the mesocortical system (Stalnaker et al., 2015). However, further studies are needed to examine the effects of CarbW ingestion.

## 5 Limitation

We recognize that the observed baseline variability is a limitation of this study. These differences could complicate the interpretation of post-water ingestion results. Future studies should consider increasing sample sizes or utilizing within-subject controls to reduce the impact of baseline variability and improve the reliability of the findings.

## Data Availability

The raw data supporting the conclusions of this article will be made available by the authors without undue reservation.

## References

[ref1] Barry E. Regan J. (2021). An examination into the effect of genetic taste status and intensity of carbonation on swallowing and palatability in healthy young adults. Int. J. Lang. Commun. Disord. 56, 812–825. doi: 10.1111/1460-6984.12629, PMID: 34125468

[ref2] Breiter H. C. Aharon I. Kahneman D. Shizgal P. (2001). Functional imaging of neural responses to expectancy and experience of monetary gains and losses. Neuron 30, 619–639. doi: 10.1016/S0896-6273(01)00303-8, PMID: 11395019

[ref3] Daw N. O'Doherty J. Dayan P. Seymour B. Dolan R. J. (2006). Cortical substrates for exploratory decisions in humans. Nature 441, 876–879. doi: 10.1038/nature04766, PMID: 16778890 PMC2635947

[ref4] Fujii N. Kataoka Y. Lai Y. F. Shirai N. Hashimoto H. Nishiyasu T. (2022). Ingestion of carbonated water increases middle cerebral artery blood velocity and improves mood states in resting humans exposed to ambient heat stress. Physiol. Behav. 255:113942. doi: 10.1016/j.physbeh.2022.113942, PMID: 35964802

[ref5] Kahnt T. Heinzle J. Park S. Q. Haynes J. D. (2010). The neural code of reward anticipation in human orbitofrontal cortex. PNAS 107, 6010–6015. doi: 10.1073/pnas.0912838107, PMID: 20231475 PMC2851854

[ref6] Kim H. Shimojo S. O’Doherty J. P. (2006). Is avoiding an aversive outcome rewarding? Neural substrates of avoidance learning in the human brain. PLoS Biol. 4, e233–e1461. doi: 10.1371/journal.pbio.0040233, PMID: 16802856 PMC1484497

[ref7] Larsson V. Torisson G. Bülow M. Londos E. (2017). Effects of carbonated liquid on swallowing dysfunction in dementia with Lewy bodies and Parkinson’s disease dementia. Clin. Interv. Aging 12, 1215–1222. doi: 10.2147/CIA.S140389, PMID: 28848329 PMC5557100

[ref8] Michou E. Mastan A. Ahmed S. Mistry S. Hamdy S. (2012). Examining the role of carbonation and temperature on water swallowing performance: a swallowing reaction-time study. Chem. Senses 37, 799–807. doi: 10.1093/chemse/bjs061, PMID: 22843761

[ref9] Min H. S. Shin H. Yoon C. H. Lee E. S. Oh M. K. Lee C. H.. (2022). Effects of carbonated water concentration on swallowing function in healthy adults. Dysphagia 37, 1550–1559. doi: 10.1007/s00455-022-10420-w, PMID: 35175420

[ref10] Miura Y. Morita Y. Koizumi H. Shingai T. (2009). Effects of taste solutions, carbonation, and cold stimulus on the power frequency content of swallowing submental surface electromyography. Chem. Senses 34, 325–331. doi: 10.1093/chemse/bjp005 19221127

[ref11] Morishita M. Mori S. Yamagami S. Mizutani M. (2014). Effect of carbonated beverages on pharyngeal swallowing in young individuals and elderly inpatients. Dysphagia 29, 213–222. doi: 10.1007/s00455-013-9493-6, PMID: 24170038

[ref12] O'Doherty J. P. Deichmann R. Critchley H. D. Dolan R. J. (2002). Neural responses during anticipation of a primary taste reward. Neuron 33, 815–826. doi: 10.1016/S0896-6273(02)00603-7, PMID: 11879657

[ref13] Plassmann H. O'Doherty J. Shiv B. Rangel A. (2008). Marketing actions can modulate neural representations of experienced pleasantness. PNAS 105, 1050–1054. doi: 10.1073/pnas.0706929105, PMID: 18195362 PMC2242704

[ref14] Roper S. D. (2014). TRPs in taste and chemesthesis. Handb. Exp. Pharmacol. 223, 827–871. doi: 10.1007/978-3-319-05161-1_5, PMID: 24961971 PMC4667542

[ref15] Sdravou K. Walshe M. Dagdilelis L. (2012). Effects of carbonated liquids on oropharyngeal swallowing measures in people with neurogenic dysphagia. Dysphagia 27, 240–250. doi: 10.1007/s00455-011-9359-8, PMID: 21822745

[ref16] Simons C. T. Klein A. H. Carstens E. (2019). Chemogenic subqualities of mouthfeel. Chem. Senses 44, 281–288. doi: 10.1093/chemse/bjz016, PMID: 31039245 PMC6538946

[ref17] Stalnaker T. A. Cooch N. K. Schoenbaum G. (2015). “What the orbitofrontal cortex does not do” in Nature neuroscience, vol. 18 (Springer Science and Business Media LLC), 620–627. 25919962 10.1038/nn.3982 PMC5541252

[ref18] Tom S. M. Fox C. R. Trepel C. Poldrack R. A. (2007). The neural basis of loss aversion in decision-making under risk. Science 315, 515–518. doi: 10.1126/science.1134239, PMID: 17255512

[ref19] Tsuji K. Tsujimura T. Sakai S. Suzuki T. Yoshihara M. Nagoya K.. (2020). Involvement of capsaicin-sensitive nerves in the initiation of swallowing evoked by carbonated water in anesthetized rats. Am. J. Physiol. Gastrointest. Liver Physiol. 319, G564–G572. doi: 10.1152/ajpgi.00233.2020, PMID: 32878469

[ref20] Walter H. Abler B. Ciaramidaro A. Erk S. (2005). Motivating forces of human actions: neuroimaging reward and social interaction. Brain Res. Bull. 67, 368–381. doi: 10.1016/j.brainresbull.2005.06.016 16216683

[ref21] Yamada T. Umeyama S. Matsuda K. (2012). Separation of fNIRS signals into functional and systemic components based on differences in hemodynamic modalities. PLoS One 7:e50271. doi: 10.1371/journal.pone.0050271, PMID: 23185590 PMC3501470

